# Interpretable LightGBM model with SHAP analysis predicts non‐excellent response to initial radioiodine therapy in differentiated thyroid carcinoma

**DOI:** 10.1002/acm2.70555

**Published:** 2026-03-27

**Authors:** Zhaoxia Luo, Yangyang Lei, Qing Zhang

**Affiliations:** ^1^ Department of Nuclear Medicine The First Affiliated Hospital Jiangxi Medical College Nanchang University Nanchang China

**Keywords:** differentiated thyroid carcinoma, machine learning, predictive modeling, radioiodine therapy, SHAP interpretability

## Abstract

**Purpose:**

To identify independent determinants influencing therapeutic outcomes of initial radioactive iodine (^1^ ^3^ ^1^I) therapy in differentiated thyroid carcinoma (DTC) and establish an interpretable predictive framework for clinical decision‐making.

**Materials and methods:**

A retrospective cohort of 950 treatment‐naïve DTC patients undergoing primary ^1^ ^3^ ^1^I therapy was randomly allocated into training (*n* = 664) and testing (*n* = 286) cohorts. Multivariable logistic regression (LR) analysis identified response‐associated variables, followed by the development of seven machine learning (ML) architectures including decision trees (DT), LR, random forests (RF), support vector machines (SVM), adaptive boosting (AdaBoost), eXtreme gradient boosting (XGBoost), and light gradient boosting machine (LightGBM). Model performance was systematically evaluated through ROC curves (AUC), calibration plots, and decision curve analysis (DCA), complemented by SHapley Additive exPlanationgs (SHAP) interpretability framework for optimal model explanation.

**Results:**

Multivariable analysis demonstrated age, ^1^ ^3^ ^1^I therapeutic interval (TI), tumor multiplicity, maximum tumor diameter (MTD), lymph node metastasis (LNM) topography, stimulated thyroglobulin (sTg), thyroglobulin antibody (TgAb) levels, administered activity (AA), and post‐therapy whole‐body scan (Rx‐WBS) findings as independent predictors of non‐excellent response (N‐ER).The LightGBM architecture achieved superior predictive accuracy (AUC = 0.896) in the testing cohort, outperforming conventional LR (AUC = 0.842).SHAP interpretation identified sTg (mean absolute SHAP value = 1.285), TgAb (mean absolute SHAP value = 0.642), and LNM topography (mean absolute SHAP value = 0.308) as principal predictive determinants. DCA confirmed that the model had a net benefit higher than the “full treatment” and “no treatment” strategies at multiple threshold probabilities, indicating its certain application value in clinical decision‐making.

**Conclusion:**

The developed LightGBM framework precisely predicts primary ^1^ ^3^ ^1^I therapeutic efficacy in DTC patients, with SHAP‐driven elucidation of clinical risk factor contributions enabling personalized therapeutic paradigms.Future integration of multicenter prospective data and molecular biomarkers is required to enhance model generalizability.

## 1 INTRODUCTION

Differentiated thyroid cancer (DTC) is the most prevalent endocrine malignancy, with an increasing global incidence over the past decades.^1^ Although overall outcomes are favorable, a clinically meaningful subset of patients fails to achieve an excellent response (ER) after initial radioiodine (^1^ ^3^ ^1^I) therapy, resulting in persistent disease, recurrence, or progression toward iodine‐refractory disease.^2^, ^3^ Early identification of patients at risk of non‐excellent response (N‑ER) is therefore important for tailoring post‐therapy management, including consideration of repeat radioiodine treatment, intensified surveillance, or timely transition to alternative systemic strategies.^4^ However, commonly used stratification systems such as the American Thyroid Association (ATA) risk framework and the Tumor–Node–Metastasis (TNM) staging system primarily reflect preoperative and pathological information and may not fully capture post‐treatment functional characteristics that influence response.

Multiple determinants of ^1^ ^3^ ^1^I therapy response have been reported, including maximum tumor diameter (MTD), multifocality, lymph node metastasis (LNM), postoperative thyroglobulin (Tg), and radioiodine avidity.^5^ In addition, biochemical and imaging markers—such as stimulated thyroglobulin (sTg), Tg antibodies (TgAb), and post‐therapy whole‐body scan (Rx‑WBS) findings—provide complementary information on residual disease burden and recurrence risk.^6^, ^7^ From a medical physics perspective, these post‐therapy imaging findings also serve as clinically available surrogates for the spatial distribution of administered activity (AA) and, indirectly, the likelihood of therapeutic exposure, particularly when patient‐specific absorbed‐dose estimates are not obtainable. Nevertheless, prior prediction approaches often evaluate factors in isolation or with limited ability to accommodate nonlinear interactions, and no widely adopted model currently integrates heterogeneous clinical, biochemical, and post‐therapy imaging information into an individualized, quantitatively interpretable risk estimate.

Machine learning (ML) methods offer a practical way to model complex, potentially nonlinear associations among multi‐domain predictors, and have shown promise for oncologic risk prediction.^8^ Existing machine‐learning studies in thyroid cancer have predominantly targeted downstream outcomes such as recurrence or distant metastasis, rather than early response after the initial ^1^ ^3^ ^1^I therapy, which represents an earlier and potentially more actionable time point for treatment tailoring and follow‐up planning.^9^, ^10^, ^11^, ^12^ For example, Schindele et al. developed an XGBoost model to predict recurrence, while Hou et al. and Sutradhar et al. focused on distant metastasis prediction and general diagnostic modeling, respectively.^10^, ^11^, ^12^ In contrast, response prediction aims to support decisions made soon after the first ^1^ ^3^ ^1^I administration; therefore, model interpretability is critical for clinical adoption. We used SHapley Additive exPlanations (SHAP) to provide both global and patient‐level explanations for tree‐based ensembles; compared with Local Interpretable Model‐agnostic Explanations (LIME), SHAP is often preferred for tree ensembles due to its theoretical consistency and more stable attributions.^13^, ^14^ However, clinical translation of ML in thyroid cancer remains challenging. Small cohorts increase the risk of overfitting and limit generalizability.^15^ Workflow and assay heterogeneity, along with missing data and limited harmonization, can compromise robustness and reproducibility.^16^ In addition, limited interpretability remains a barrier to clinical acceptance and integration into established decision‐making frameworks.^17^

Therefore, this study aims to systematically identify independent predictors of N‑ER following initial ^1^ ^3^ ^1^I therapy in DTC patients and to develop a clinically interpretable LightGBM model augmented with SHAP analysis for personalized risk stratification. By integrating pathological variables, biochemical markers, and post‑therapy imaging–derived indicators available in routine practice, we seek to provide an explainable, data‑driven framework that improves individualized risk assessment and supports tailored management of DTC patients undergoing radioiodine therapy.

## 2 MATERIALS AND METHODS

### 2.1 Patient data

We conducted a retrospective cohort study of 950 consecutive DTC patients undergoing postoperative radioactive iodine (RAI) therapy with completed follow‐up at our tertiary referral center from October 2020 to March 2024. The study population comprised 656 males (69.1%) and 294 females (30.9%) with a median age of 42 years (IQR 28–52). A computer‐generated random sequence allocated 70% of the patients (*n* = 664) to the training set and 30% (*n* = 286) to the testing set. The training set was used for model development with 5‐fold cross‐validation to optimize hyperparameters and reduce overfitting. Eligibility criteria required: (1) Total/near‐total thyroidectomy ± neck lymph node dissection; (2) Histologically confirmed DTC per WHO classification; (3) Completion of initial RAI therapy at our center; (4) ≥6 months' structured follow‐up post‐RAI. Exclusion criteria encompassed: (1) Incomplete therapeutic response assessment data; (2) Missing > 20% predefined essential variables per case report form. TNM staging was determined according to the American Joint Committee on Cancer (AJCC) 7th edition criteria.^18^ Based on postoperative histopathology reports, the topographic extent of lymph node metastasis (LNM topography) was categorized as: no metastasis (pN0), central (Level VI), lateral, or others. Following thyroidectomy with cervical lymphadenectomy, RAI therapy (3.7‐9.25 GBq) was administered 2–4 weeks after levothyroxine withdrawal, with dose determination guided by TNM staging, postoperative biochemical (sTg) and imaging findings, combined with individualized clinical considerations. Post‐therapy ^1^ ^3^ ^1^I whole‐body scintigraphy with SPECT/CT was acquired 48–72 h after RAI administration using a hybrid gamma camera (Intevo Bold, Siemens Healthineers, Erlangen, Germany) with standardized acquisition protocols (matrix size 256 × 1024, energy window 364 ± 20 keV, high‐energy general‐purpose collimator, scan speed 5 cm/min). The findings on Rx‐WBS were categorized as showing no specific uptake, locoregional lymph node uptake, or distant metastasis uptake, and this variable was used in subsequent predictive modeling. Additionally, the dominant residual or metastatic focus was assigned a semiquantitative uptake score (0–2), where 0 indicated no abnormal uptake above background, 1 indicated uptake lower than the physiological nasopharyngeal background, and 2 indicated uptake equal to or higher than this background. Risk‐adapted thyroid stimulating hormone (TSH) suppression therapy (starting dose 2.02.5 µg/kg/day) was initiated 24–48 h post‐RAI, with target TSH levels stratified by ATA recurrence risk categories: < 0.1 mIU/L (high‐risk), 0.10.5 mIU/L (intermediate‐risk), and 0.52.0 mIU/L (low‐risk).^19^ Standardized surveillance included: (1) Biochemical assessments (TSH, Tg, TgAb, free triiodothyronine (FT3), free thyroxine (FT4)) performed at approximately 6 weeks after ^1^ ^3^ ^1^I therapy and subsequently at 2‑month intervals; (2) Neck ultrasonography at 4‐month intervals; (3) Diagnostic ^1^ ^3^ ^1^I SPECT/CT with stimulated Tg measurement after 3‐week levothyroxine withdrawal at 6–9 months; (4) Semiannual chest CT for high‐risk patients.

### 2.2 Efficacy grouping

Therapeutic responses were classified according to the 2015 ATA criteria:^19^ ER was defined as (1) absence of structural disease on imaging, (2) suppressed Tg < 0.2 ng/mL, and (3) sTg < 1 ng/mL. Indeterminate Response (IDR) included: (1) nonspecific imaging abnormalities (e.g., faint thyroid bed uptake on diagnostic whole‐body scan [Dx‐WBS]) with (2) stable/decreasing TgAb titers, (3) suppressed Tg 0.21 ng/mL, and/or (4) sTg 1–10 ng/mL. Biochemical Incomplete Response (BIR) required: (1) no structural evidence on imaging, (2) rising TgAb levels (> 20% increase over baseline), (3) suppressed Tg ≥1 ng/mL, or (4) sTg ≥10 ng/mL. Structural Incomplete Response (SIR) was characterized by (1) imaging‐confirmed persistent/recurrent disease (locoregional or distant metastasis) irrespective of (2) serum Tg/TgAb concentrations. All imaging findings underwent blinded independent review by two board‐certified nuclear radiologists, with final outcomes categorized as ER versus N‐ER (composite of IDR, BIR, SIR).

### 2.3 Data preprocessing

After applying the patient‐level exclusion criteria, variables with more than 10% missing values across the remaining cohort were excluded. Specifically, the following variables were excluded due to missingness exceeding 10%: (1) the specific anatomical location of thyroid nodules, and (2) genetic mutation status, which were not consistently recorded in the clinical database. Missing data in retained variables were imputed via multiple imputation by chained equations (MICE) using the mice package (v3.14.0) with 10 imputation cycles.^20^ Continuous variables were standardized using Z‐score transformation (mean = 0, standard deviation [SD] = 1), and categorical variables were one‐hot encoded. The semiquantitative uptake score was treated as an ordinal three‐level variable in these additional analyses and was not used as an input feature in the prediction models.

### 2.4 Predictive modeling

Multivariable LR identified independent predictors (α = 0.05), with results visualized via nomogram using the rms package (v6.3‐0). Seven ML models were developed: decision tree (DT, rpart v4.1‐16), LR (LR, glm v4.1‐0), random forest (RF, randomForest v4.7‐1.1), support vector machine (SVM, e1071 v1.7‐12), adaptive boosting (AdaBoost, adabag v4.2), eXtreme gradient boosting (XGBoost, xgboost v1.6.0.1), and light gradient boosting machine (LightGBM, lightgbm v3.3.5). Hyperparameter tuning used 5‐fold cross‐validated grid search (caret v6.0‐93). The final LightGBM model used the hyperparameters selected via grid search with five‐fold cross‐validation: a learning rate of 0.05, a maximum tree depth of 5, a maximum number of leaves of 31, a feature fraction of 0.8, and a bagging fraction of 0.8, and 100 boosting iterations. The grid search explored the following hyperparameter space: learning rate ∈ {0.01, 0.03, 0.05, 0.1}; maximum tree depth ∈ {3, 4, 5, 6}; boosting iterations ∈ {50, 100, 150, 200}; number of leaves ∈ {16, 31, 63}; feature_fraction ∈ {0.6, 0.8, 1.0}; and bagging_fraction ∈ {0.6, 0.8, 1.0}.

### 2.5 Model evaluation

Performance metrics included: area under the receiver operating characteristic (ROC) curve (AUC), accuracy (ACC), sensitivity (SEN), specificity (SPC), positive predictive value (PPV), negative predictive value (NPV), F1‐score, and Youden index (YI). ROC curves summarize the tradeoff between true positive and false positive rates across thresholds. The Youden index (YI), defined as sensitivity + specificity – 1, was used to determine the optimal threshold for binary classification. Calibration performance was assessed using the calibration slope and intercept, where the slope indicates model overfitting (ideal = 1) and the intercept measures systematic bias (ideal = 0). Calibration curves were generated using locally estimated scatterplot smoothing (LOESS) with span = 0.75. LOESS is a nonparametric regression method that fits local polynomial curves to visualize calibration. Clinical utility was evaluated using decision curve analysis (DCA), which calculates net benefit by comparing true‐positive and false‐positive outcomes across a range of threshold probabilities. Bootstrap resampling (1000 replicates) was used to compute 95% percentile confidence intervals (CIs) for all performance metrics.

### 2.6 Interpretability framework

SHAP values were computed using shapviz (v0.9.0) package in R. SHAP values quantify the marginal contribution of each feature to the model prediction in log‐odds space.^21^ A positive SHAP value increases the predicted risk of N‐ER, while a negative value reduces it. Visualization included: (1) a waterfall plot to explain an individual prediction by showing how each feature shifts the model output from the baseline to the final prediction; and (2) a beeswarm (summary) plot to display the distribution and direction of SHAP values across all samples, with features ranked by mean absolute SHAP values to reflect global importance.

### 2.7 Statistical analysis

All analyses were performed in R (version 4.1.0; R Foundation for Statistical Computing). Categorical variables were compared using Pearson's chi‑squared test with Yates’ continuity correction, and continuous variables were compared using the Mann–Whitney U test. Spearman's rank correlation coefficient was used for correlations between continuous variables. For the semiquantitative uptake score, N‑ER rates across categories were compared using Pearson's chi‑squared test with exact binomial 95% CIs, and sTg levels were compared using the Kruskal–Wallis rank‑sum test. A two‑sided *P* value < 0.05 was considered statistically significant.

## 3 RESULTS

### 3.1 General information comparison

**FIGURE 1:**
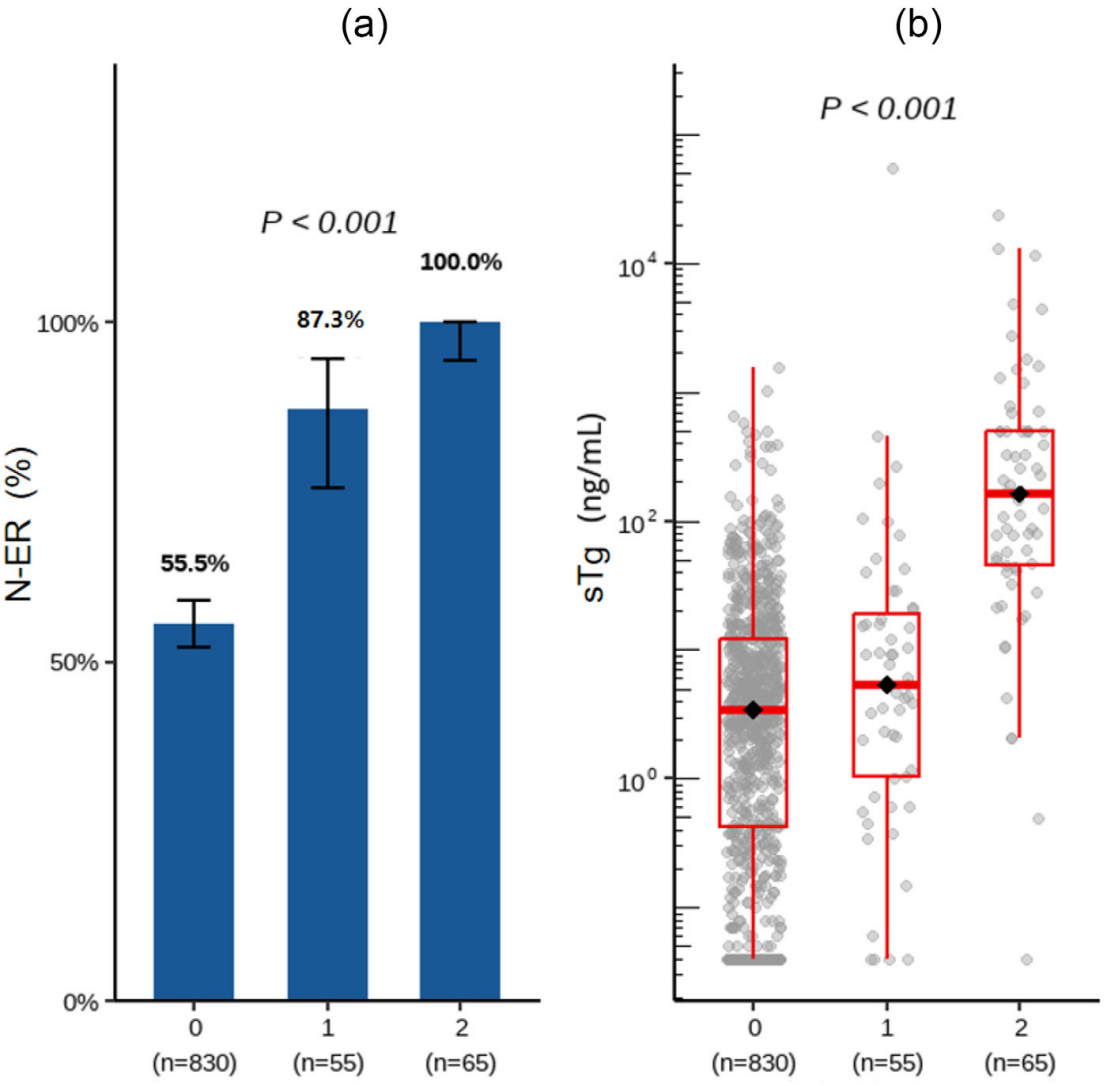
Association of post‑therapy radioiodine uptake score with N‐ER and sTg. (a) The association between uptake score (0–2) and non‑excellent response (N‑ER) was evaluated using the Pearson chi‑squared test. (b) Distributions of stimulated thyroglobulin (sTg) concentrations (linear scale) across uptake score groups were compared using the Kruskal–Wallis test.

Among the 950 enrolled DTC patients, 376 (39.6%) achieved ER and 574 (60.4%) were classified as N‑ER (Table 1). Significant between‑group differences were observed in therapeutic interval (TI), tumor multifocality, LNM topography, neck ultrasound findings, chest CT results, Rx‑WBS patterns, age, MTD, MLD, sTg, TgAb levels, and AA (all *P* < 0.05). No significant differences were found in sex, tumor bilaterality, pathological type, TSH levels, or anthropometric indices including height, weight, and BMI (all *P* > 0.05). When stratified by the semiquantitative uptake score, the N‑ER rate increased across categories, being 55.5% for score 0 (461/830; 95% CI 52.1%–59.0%), 87.3% for score 1 (48/55; 95% CI 75.5%–94.7%), and 100.0% for score 2 (65/65; 95% CI 94.5%–100.0%). The overall difference was significant (Pearson *χ* ^2^ = 67.42, *P* < 0.001). Median sTg levels also differed across uptake score groups, being 3.45 ng/mL (IQR 0.42–11.95), 5.38 ng/mL (IQR 1.02–19.20), and 161.80 ng/mL (IQR 44.60–500.00), respectively (Kruskal–Wallis H = 121.16, *P* < 0.001) (Figure 1).

**TABLE 1:** Baseline characteristics in DTC patients (N‐ER vs ER).

CHARACTERISTIC	N‐ER(*n* = 574,60%)	ER(*n* = 376,40%)	*P*‐value
Sex(*n*%)			0.249
Male	186(32%)	108(29%)	
Female	388(68%)	268(71%)	
Therapeutic interval(*n*%)			**0.008**
≤3 months	368(64%)	274(73%)	
>3 months	206(36%)	102(27%)	
Tumor bilaterality(*n*%)			0.162
No	222(39%)	163(43%)	
Yes	352(61%)	213(57%)	
Tumor multifocality(*n*%)			**0.008**
Single spot	223(39%)	179(48%)	
Multiple spots	351(61%)	197(52%)	
LNM topography(*n*%)			**<**0.001
No	29(5%)	43(11%)	
Central neck	158(28%)	173(46%)	
Lateral neck	339(59%)	150(40%)	
Others	48(8%)	10(3%)	
Pathological type(*n*%)			0.149
Classical	515(90%)	335(89%)	
Nonclassical	44(8%)	37(10%)	
Follicular carcinoma	15(2%)	4(1%)	
Neck ultrasound(*n*%)			**<0.001**
No	485(84%)	353(94%)	
yes	89(16%)	23(6%)	
Chest CT(*n*%)			**<0.001**
No	548(95%)	376(100%)	
Yes	26(5%)	0(0%)	
Rx‐WBS(*n*%)			**<0.001**
No	459(80%)	367(98%)	
LNM	81(14%)	8(2%)	
DM^1^	34(6%)	1(0%)	
Age(years),Median(IQR)	38(30,49)	43(33,51)	**<0.001**
MTD(cm),Median(IQR)	1.6(1.20,2.50)	1.5(1,2)	**<0.001**
MLD(cm),Median(IQR)	0.8(0.60,1.10)	0.8(0.30,0.90)	**<0.001**
TSH(mIU/L),Median(IQR)	82.1(61.1,100.00)	80.025(60.7,100.00)	0.297
sTg(ng/mL),Median(IQR)	9.375(2.43,36.96)	1.615(0.32,4.07)	**<0.001**
TgAb(IU/mL),Median(IQR)	16.65(12.89,73.98)	16.5(12.76,25.23)	**0.012**
Weight(kg),Median(IQR)	61.25(55,70)	60.8(55,70)	0.934
Height(cm),Median(IQR)	160(157,167)	160(157,165)	0.156
BMI^2^ (kg/m^2^),Median(IQR)	23.807(21.44,26.04)	23.828(21.54,26.35)	0.578
AA(mCi),Median(IQR)	160(150,180)	150(150,150)	**<0.001**

^1^ DM = distant metastasis.

^2^ BMI = body mass index.

### 3.2 Cohort stratification

The 950 DTC patients were randomly stratified into training and testing cohorts, with balanced baseline characteristics between groups (Table 2). All baseline variables showed comparable distributions between cohorts (all *P* > 0.05) except for LNM topography (*P* < 0.001).

**TABLE 2:** The distribution of variables in the training and testing sets.

Characteristic	Training set (*n* = 664,70%)	Testing set (*n* = 286,30%)	*P*‐value
Efficacy(*n*%)			1
N‐ER	401(60%)	173(60%)	
ER	263(40%)	113(40%)	
Sex(*n*%)			1
Male	205(31%)	89(31%)	
fFmale	459(69%)	197(69%)	
Therapeutic interval(*n*%)			1
≤3 months	449(68%)	193(67%)	
>3 months	215(32%)	93(33%)	
Tumor bilaterality(*n*%)			0.624
No	273(41%)	112(39%)	
Yes	391(59%)	174(61%)	
Tumor multifocality(*n*%)			0.940
Single spot	282(42%)	120(42%)	
Multiple spots	382(58%)	166(58%)	
LNM topography(*n*%)			**<0.001**
No	46(7%)	26(9%)	
Central	222(33%)	109(38%)	
Lateral	352(53%)	137(48%)	
Others	44(7%)	14(5%)	
Pathological type(*n*%)			0.332
Classical	590(89%)	260(91%)	
Nonclassical	62(9%)	19(7%)	
Follicular carcinoma	12(2%)	7(2%)	
Neck ultrasound(*n*%)			0.962
No	585(88%)	253(88%)	
Yes	79(12%)	33(12%)	
Chest CT(*n*%)			0.313
No	643(97%)	281(98%)	
Yes	21(3%)	5(2%)	
Rx‐WBS(*n*%)			0.857
No	579(87%)	247(86%)	
LNM	62(9%)	27(10%)	
DM	23(4%)	12(4%)	
Age(years),Median(IQR)	40.5(32,51.00)	40(31.25,49.00)	0.518
MTD(cm),Median(IQR)	1.5(1.10,2.50)	1.5(1.10,2.28)	0.582
MLD(cm),Median(IQR)	0.8(0.50,1.00)	0.8(0.40,1.00)	0.365
TSH(mIU/L),Median(IQR)	80.3(59.15,100)	84.85(66.22,100.00)	0.131
sTg(ng/mL),Median(IQR)	4.31(0.53,16.33)	3.88(0.61,15.08)	0.670
TgAb(IU/mL),Median(IQR)	16.465(12.80,42.33)	16.85(12.94,31.58)	0.873
Weight(kg),Median(IQR)	62(55,70)	60(55.00,70)	0.320
Height(cm),Median(IQR)	160(157,166)	160(157,167)	0.723
BMI(kg/m^2^),Median(IQR)	23.828(21.48,26.27)	23.577(21.48,26.15)	0.804
AA(mCi),Median(IQR)	150(150,180)	150(150,160)	0.504

### 3.3 Multivariable LR analysis

**FIGURE 2:**
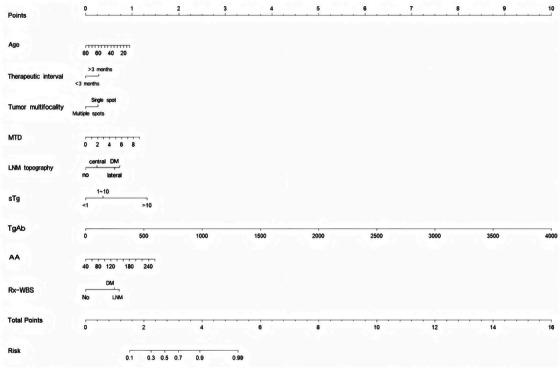
Nomogram for predicting the probability of N‐ER to initial ^1^ ^3^ ^1^I therapy in patients with DTC. The model integrates independent predictive variables including age (years), TI (months), tumor multifocality, MTD(cm), LNM topography, sTg (ng/mL), TgAb (IU/mL), AA (mCi), and Rx‐WBS findings. To estimate the risk of N‐ER, locate each variable on its axis, draw a vertical line to the “Points” row to determine the score for each factor, sum the points, and project the total score onto the “Risk” scale to obtain the predicted probability.

Multivariable LR analysis identified eight statistically significant independent predictors of N‐ER after initial ^1^ ^3^ ^1^I therapy in DTC patients: age (odds ratio [OR] = 0.97, *p* = 0.002), TI (OR = 1.60, *p* = 0.031), tumor multifocality (OR = 1.57, *p* = 0.024), LNM topography (OR = 1.84, *p* < 0.001), sTg (OR = 1.08, *p* < 0.001), TgAb (OR = 1.004, *p* < 0.001), AA (OR = 1.01, *p* = 0.014), and Rx‐WBS findings (OR = 2.55, *p* = 0.024). MTD (OR = 1.24, *p* = 0.051) showed a borderline association and was retained in the final model. These variables were integrated into a clinically applicable nomogram (Figure 2) to facilitate individualized estimation of N‐ER risk.

Subgroup analyses were performed for TI, tumor multifocality, LNM topography, and sTg (Table 3). Delayed therapy (> 3 months) was associated with higher N‑ER risk (OR = 2.03, 95% CI 1.53–2.70), as were multifocality (OR = 1.75, 95% CI 1.42–2.15), lateral neck metastasis (OR = 2.35, 95% CI 1.87–2.96), and other metastatic sites (OR = 4.50, 95% CI 2.09–9.68). sTg > 10 ng/mL showed the strongest association (OR = 8.13, 95% CI 5.32–12.42). All subgroup comparisons were significant (*P* < 0.001).

**TABLE 3:** Multivariable LR subgroup analysis.

Group	Proportion(*n*%)	OR(95%CI)	*P*‐value
Therapeutic interval(*n*%)			
≤3 months	257/449(57.2%)	1.339(1.111.61)	0.002
>3 months	144/215(67.0%)	2.028(1.532.70)	**<0.001**
Tumor multifocality(*n*%)			
Single spot	158/282(56.0%)	1.274(1.011.61)	0.043
Multiple spots	243/382(63.6%)	1.748(1.422.15)	**<0.001**
LNM topography(*n*%)			
No	18/46(39.1%)	0.643(0.361.16)	0.143
Central	100/222(45.0%)	0.82(0.631.07)	0.14
Lateral	247/352(70.2%)	2.352(1.872.96)	**<0.001**
Others	36/44(81.8%)	4.5(2.099.68)	**<0.001**
sTg(*n*%)			
<1	323/579(45.1%)	0.822(0.621.09)	0.174
110	118/250(47.2%)	0.894(0.701.15)	0.376
>10	195/219(89.0%)	8.125(5.3212.42)	**<0.001**

### 3.4 Predictive model development

**FIGURE 3:**
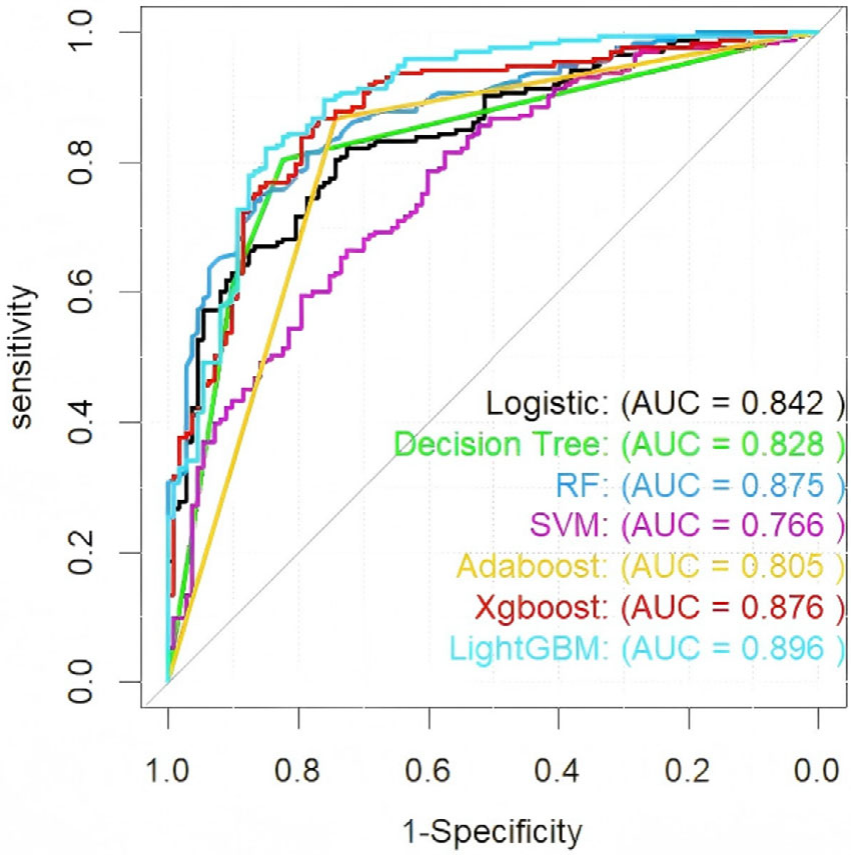
ROC curves of seven machine learning models in the testing cohort for predicting N‐ER to initial ^1^ ^3^ ^1^I therapy in patients with DTC. The models include LR (AUC = 0.842), DT (AUC = 0.828), RF (AUC = 0.875), SVM (AUC = 0.766), AdaBoost (AUC = 0.805), XGBoost (AUC = 0.876), and LightGBM (AUC = 0.896). Among these, LightGBM achieved the highest area under the curve in the validation set, indicating its superior generalization ability and predictive performance.

**FIGURE 4:**
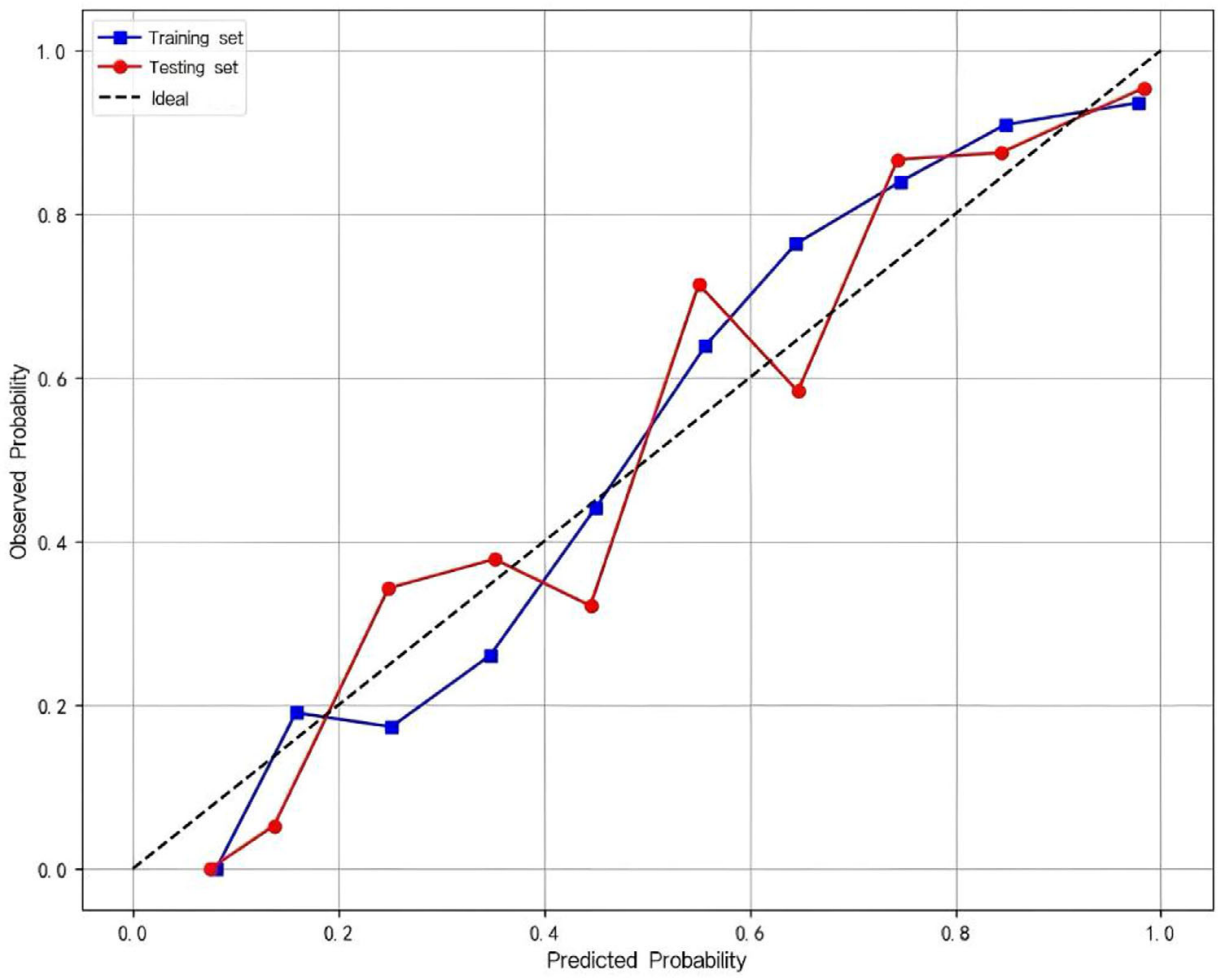
Calibration curves of the LightGBM model for predicting N‐ER to initial ^1^ ^3^ ^1^I therapy in patients with DTC. The blue line represents the training set and the red line represents the testing set. The dashed diagonal line indicates perfect calibration, where predicted probabilities align exactly with observed outcomes. The proximity of the calibration curves to the ideal line in both cohorts suggests good agreement between predicted and actual probabilities, demonstrating strong model calibration.

**FIGURE 5:**
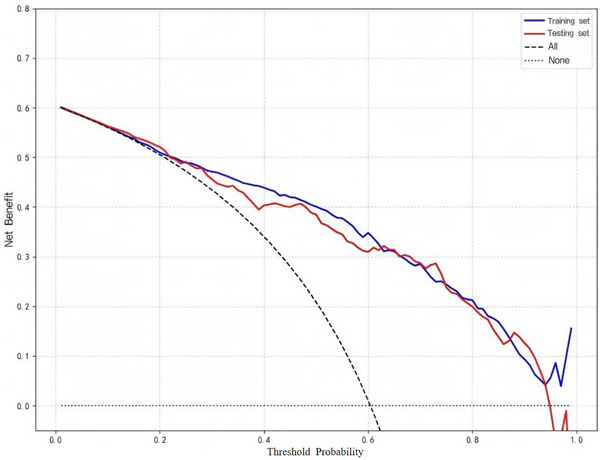
DCA of the LightGBM model for predicting N‐ER to initial ^1^ ^3^ ^1^I therapy in patients with DTC. The blue and red lines represent the net clinical benefit across a range of threshold probabilities in the training and testing cohorts, respectively. The black dashed line (“All”) assumes all patients receive ^1^ ^3^ ^1^I therapy; its net benefit at each threshold probability Pt is calculated as (sensitivity—(1 – specificity) × Pt / (1 – Pt)) using the overall event rate in the cohort. The dotted line (“None”) assumes no patients are treated.

Seven ML models (DT, LR, RF, SVM, AdaBoost, XGBoost, and LightGBM) incorporating the nine identified predictors were evaluated. Model performance metrics (ACC, SEN, SPC, PPV, NPV, F1‐score, and YI) are summarized in Table 4. Overall, all models demonstrated discrimination in both the training and testing sets, with training AUCs ranging from 0.799 (AdaBoost) to 1.000 (XGBoost) and testing AUCs ranging from 0.766 (SVM) to 0.896 (LightGBM) (Figure 3). XGBoost achieved the highest training AUC (1.000; 95% CI: 0.999–1.000) but a lower AUC on the testing set (0.876; 95% CI: 0.834–0.918) than LightGBM, suggesting possible overfitting. By contrast, LightGBM showed strong performance on the testing set and better generalization; therefore, it was selected as the final model for subsequent analyses. For the selected LightGBM model, calibration curves showed good agreement between predicted probabilities and observed outcomes (Figure 4). DCA demonstrated a higher net benefit than the “treat‐all” and “treat‐none” strategies across a range of threshold probabilities (Figure 5).

**TABLE 4:** Evaluation metrics of various models in the training and testing set.

		ACC	AUC	SEN	SPC	PPV	NPV	F1	YI
Training set	DT	0.797	0.800	0.815	0.768	0.842	0.732	0.829	0.584
	LR	0.792	0.863	0.810	0.764	0.840	0.726	0.825	0.575
	RF	0.905	0.964	0.943	0.848	0.904	0.907	0.923	0.791
	SVM	0.801	0.887	0.848	0.730	0.827	0.759	0.837	0.578
	Adaboost	0.818	0.799	0.890	0.707	0.823	0.809	0.855	0.597
	XGBoost	0.997	1.000	0.998	0.996	0.998	0.996	0.998	0.994
	LightGBM	0.908	0.975	0.955	0.837	0.899	0.924	0.926	0.792
Testing set	DT	0.811	0.828	0.803	0.823	0.874	0.732	0.837	0.626
	LR	0.762	0.842	0.775	0.743	0.822	0.683	0.798	0.518
	RF	0.801	0.875	0.844	0.735	0.830	0.755	0.837	0.578
	SVM	0.682	0.766	0.717	0.628	0.747	0.592	0.723	0.345
	Adaboost	0.818	0.805	0.867	0.743	0.838	0.785	0.852	0.610
	XGBoost	0.825	0.876	0.855	0.779	0.855	0.779	0.855	0.634
	LightGBM	0.825	0.896	0.844	0.796	0.864	0.769	0.854	0.640

### 3.5 Model interpretability

**FIGURE 6:**
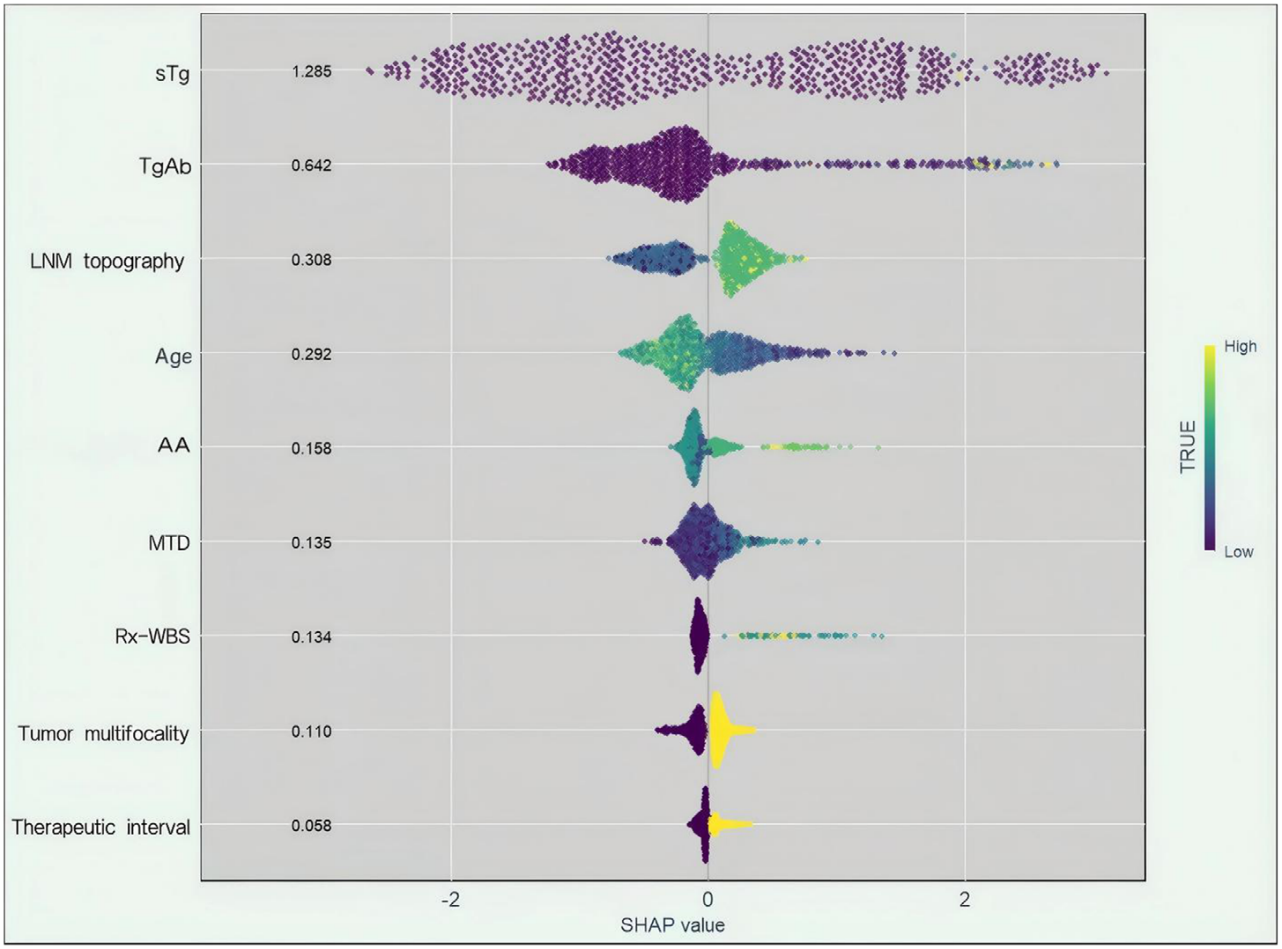
SHAP summary plot illustrating the contribution and importance of each feature in the LightGBM model for predicting N‐ER to initial ^1^ ^3^ ^1^I therapy in patients with DTC. Features are ranked from top to bottom by their mean absolute SHAP values, reflecting their overall importance in the model. sTg, TgAb, and LNM topography identified as the top contributors to the model's output. Each point represents an individual patient, with colors indicating the relative value of the corresponding feature (from low [purple] to high [yellow]). The horizontal axis reflects the SHAP value, denoting the direction and magnitude of the feature's impact on the model's prediction. Notably, the color distribution may appear concentrated even when SHAP values are close to zero, particularly in features with highly skewed or binary distributions. In such cases, the color gradient indicates input distribution rather than predictive influence.

To enhance clinical interpretability and understand the contribution of individual variables to the LightGBM model, SHAP analysis was employed. In Figure 6, the SHAP summary plot demonstrates the global distribution of SHAP values across all patients. Each dot represents one patient, with color gradients corresponding to feature values (from low to high). Features are ordered by mean absolute SHAP values, and the plot summarizes the magnitude, direction, and variability of each feature's impact. sTg was the most influential feature, with high sTg values consistently associated with strong positive SHAP values. TgAb levels also showed a clear positive relationship with N‐ER risk. Age demonstrated a notable inverse relationship: younger patients tended to have higher SHAP values, suggesting increased risk. Although AA and TI had smaller average effects, their SHAP values spanned a wider range, suggesting context‐dependent contributions. These patterns are reflected by the feature ordering in Figure 6.

**FIGURE 7:**
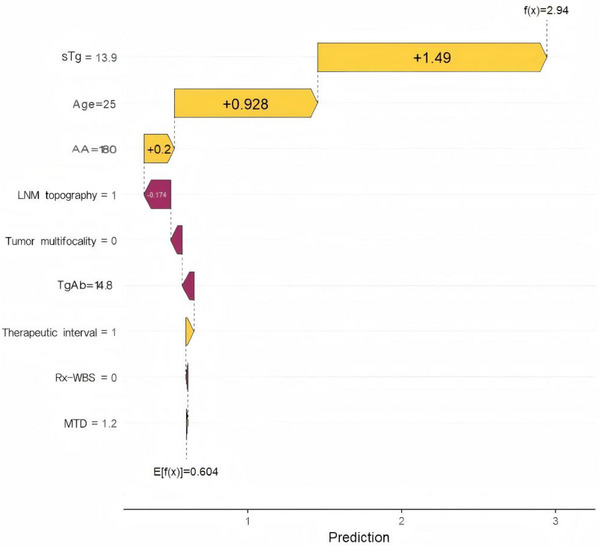
SHAP waterfall plot illustrating the contribution of each feature to the LightGBM model's prediction for a representative patient with DTC undergoing initial ^1^ ^3^ ^1^I therapy. The base value (*E*[*f*(*x*)] = 0.604) represents the mean model output across all patients, and the final prediction (*f*(*x*) = 2.94) results from the cumulative additive effects of individual features. Positive SHAP values (yellow) increase the predicted risk of N‐ER, while negative values (red) reduce it. sTg (13.9) and age (25 years) were the primary contributors to the elevated risk prediction in this case.

Figure 7 presents a SHAP waterfall plot for an individual patient, illustrating how each input variable shifts the model's prediction from the expected (baseline) model output. The base value of the model was 0.604, representing the expected model output across all patients (on the log‐odds scale). Among the features, a sTg level of 13.9 ng/mL exerted the strongest positive impact on the predicted risk of N‐ER, contributing +1.49 to the final log‐odds score. Age (25 years) also significantly increased the prediction (+0.928), followed by AA (180 mCi, +0.2). In contrast, LNM topography (coded as 1) had a modest negative contribution (−0.174), while other variables such as TgAb, MTD, and imaging findings exerted minimal influence in this specific case. The final predicted value for this patient was *f*(*x*) = 2.94, indicating a high predicted risk of N‐ER.

## 4 DISCUSSION

This study systematically identified nine independent predictors of N‐ER to initial radioiodine therapy in patients with DTC and developed a clinically interpretable LightGBM‐based ML model for individualized risk stratification. The results demonstrated robust predictive performance; among the evaluated algorithms, LightGBM showed the best discrimination on the testing set and was therefore used as the final model for subsequent analyses. The integration of SHAP analysis further enhanced model transparency by quantifying and visualizing the contribution of each feature to the predicted outcome. Consistent with the model findings, analyses based on the semiquantitative uptake score demonstrated increasing N‑ER rates and higher sTg levels across score categories. These results indicate that uptake score and sTg are clinically informative for characterizing residual disease burden and early response after radioiodine therapy.

The SHAP‐based interpretability of the LightGBM model provides a hierarchical framework for understanding the relative influence of clinical, biochemical, and imaging features on treatment response.^21^ Among all variables, sTg was consistently identified as the most influential predictor, exhibiting the highest mean absolute SHAP value in the global summary plot (1.285). Higher sTg values were associated with substantially increased SHAP contributions, indicating a monotonic relationship between pre‐therapeutic sTg levels and N‐ER risk. While this trend was observed across the cohort, it should be noted that SHAP values do not directly imply linearity in a regression sense. Rather, this finding suggests that elevated sTg levels, particularly those exceeding 10 ng/mL, are strongly associated with poor therapeutic response, which is consistent with prior studies that have proposed this threshold as indicative of biochemical persistence following total thyroidectomy and remnant ablation.^22^, ^23^ The gradient in N‐ER proportion and sTg concentration across uptake score categories supports this pattern and suggests that sTg and uptake scoring reflect complementary aspects of the same underlying disease burden.

TgAb also emerged as significant contributors to the model, with a mean SHAP value of 0.642. Although TgAb is traditionally viewed as an assay interferent that compromises the accuracy of sTg measurement,^24^, ^25^, ^26^, ^27^ our model suggests that TgAb levels themselves provide independent prognostic information. This observation supports the emerging concept that TgAb may not only reflect immune response but may also indicate the presence of residual or recurrent disease.^28^, ^29^ However, the interpretation of TgAb should be approached with caution, particularly in patients with dynamic antibody trends.^30^ While our model incorporated only baseline TgAb levels, further studies incorporating TgAb kinetics could clarify their additive value.

Lymph node topography also emerged as one of the most influential predictors in the model, as indicated by its high mean absolute SHAP value. This likely reflects the prognostic significance of anatomical patterns of lymphatic involvement in DTC. Previous studies have demonstrated that the location and extent of nodal metastases are associated with more aggressive tumor behavior, higher recurrence rates, and increased risk of distant metastasis.^31^, ^32^ In contrast, central compartment involvement alone may carry a more favorable prognosis. Therefore, the model's reliance on nodal topography aligns with established clinical knowledge and suggests that beyond the mere presence of nodal disease, the spatial distribution of metastatic involvement carries important biological and therapeutic implications. These findings underscore the value of incorporating detailed anatomical features into predictive frameworks and support the clinical relevance of SHAP‐derived feature importance in interpreting model behavior.

Age was another important predictor, with a mean SHAP value of 0.292. Notably, the SHAP analysis revealed an inverse relationship between age and N‐ER risk, where younger patients exhibited higher SHAP values and were therefore more likely to have suboptimal therapeutic responses. This observation corresponds with previous epidemiological reports indicating that younger DTC patients may have more aggressive disease phenotypes despite generally favorable survival outcomes.^33^, ^34^, ^35^ Although the present study did not include molecular profiling, prior literature has suggested that tumor biology in younger individuals may be characterized by distinct immune microenvironments, altered iodine metabolism, and unique gene expression signatures.^36^, ^37^, ^38^ These findings, while not directly evaluated in our study, provide a plausible biological rationale for the age‐related gradient observed in our model and merit further exploration in future research.

Interestingly, AA was found to have a modest but non‐negligible impact on prediction outcomes (mean absolute SHAP = 0.158). In some patients, higher AA (> 5.55 GBq) corresponded to higher SHAP values, suggesting a paradoxical pattern in which activity escalation may mark greater disease burden rather than improved treatment efficacy.^39^ Although Rx‑WBS and Dx‑WBS were available, lesion‑level dosimetry was not performed because of the retrospective design and the lack of quantitative SPECT calibration. This may reflect clinical practice, where higher activities are often preferentially prescribed to patients with more extensive or aggressive disease. While AA correlates only weakly with tumor‑absorbed dose in some settings,^40^ accumulating evidence supports absorbed dose as the key determinant of response, and recent ^1^ ^3^ ^1^I dosimetric studies in DTC have reported dose–response relationships at lesion and whole‑body levels, underscoring the value of individualized dosimetry in selected patients.^41^, ^42^ Variability in iodine uptake despite comparable AA may arise from heterogeneous NIS expression, reduced perfusion, necrosis, and volume‑related mass effects that limit tracer penetration, highlighting the limitations of using AA as a surrogate for effective radiation delivery. Future studies incorporating voxel‑based dosimetry and molecular imaging biomarkers may help clarify these mechanisms and enable more personalized treatment planning.^43^

Although MTD did not meet the conventional *p*‐value threshold for statistical significance (*p* = 0.051), we chose to retain it in the final predictive model for several reasons. First, it demonstrated a marginal association with N‐ER, suggesting a potential signal at the boundary of significance. Second, the biological plausibility of tumor size influencing iodine uptake and disease persistence supports its inclusion. Third, inclusion of MTD improved the model's overall predictive performance, as assessed by cross‐validation metrics. Lastly, interactions between MTD and other variables may capture clinically relevant patterns that are not fully reflected by *p*‐values alone. Therefore, we considered it appropriate to retain MTD in the multivariate framework to preserve both statistical robustness and clinical interpretability.

The interpretability of the model, enabled by SHAP analysis, ensures transparency and traceability, which are key requirements for regulatory compliance and for maintaining clinician trust.^44^ By quantitatively ranking feature importance, SHAP values provide actionable insights into which variables drive risk prediction. For example, clinicians may prioritize follow‐up or additional imaging in patients with high sTg and TgAb levels, particularly those who are younger or present with lateral neck metastases.^19^ This stratified approach supports precision medicine by targeting interventions to those most likely to benefit. It is worth noting, however, that despite its strong performance, the model exhibited a decrease in AUC when transitioning from the training to the testing cohort (0.975 to 0.896), suggesting potential overfitting. While this drop is within acceptable bounds, it highlights the importance of external validation in independent cohorts to ensure generalizability.^45^

This study has several limitations. Its retrospective design may introduce selection bias and residual confounding, and the findings should be validated in external cohorts and, ideally, prospectively. Individualized lesion‐ or voxel‐level dosimetry and dose–response analysis could not be performed because the historical dataset lacked the quantitative imaging and time–activity data required for absorbed‐dose calculation; thus, we used a semiquantitative post‐therapy SPECT/CT uptake stratification as a surrogate of iodine avidity and activity localization. Reproducibility in routine practice may also be influenced by inter‐institutional variability in laboratory assays (e.g., TgAb platforms) and imaging workflows, particularly Rx‐WBS timing. Although SHAP improves transparency, feature attributions should not be interpreted as causal effects.^46^ Future work should evaluate molecular/genomic biomarkers,^47^ radiomics‐based features,^48^ and real‐world implementation to determine clinical impact.

## 5 CONCLUSION

This study developed and validated a LightGBM‐based ML model for predicting N‐ER to initial radioiodine therapy in patients with differentiated thyroid carcinoma. The model demonstrated strong predictive performance and interpretability, with SHAP analysis highlighting sTg, TgAb, age, and lymph node topography as the most influential predictors. While challenges related to generalizability and standardization remain, the proposed framework represents a promising step toward personalized treatment planning in DTC. Future prospective studies incorporating molecular, radiological, and kinetic biomarkers are warranted to further refine prediction accuracy and facilitate clinical translation.

## AUTHOR CONTRIBUTIONS

**Zhaoxia Luo**: writing—the original draft; software, formal analysis. **Yangyang Lei**: investigation; data curation; conceptualization. **Qing Zhang**: supervised the study.

## FUNDING INFORMATION

This work was supported by the National Natural Science Foundation of China (grant number: 82260356 and 8226070308) and the Key Project of Natural Science Foundation of Jiangxi Province (20232ACB206026).

## CONFLICT OF INTEREST STATEMENT

The authors declare no conflicts of interest.

## ETHICS STATEMENT

This study was conducted in accordance with the principles of the Declaration of Helsinki and was approved by the institutional ethics committee (Approval No. 2024 CDYFYYLK(10‐033)).

## CONSENT STATEMENT

The authors unanimously consent to the publication of this manuscript.

## Data Availability

The datasets generated during the current study are available from the corresponding author on reasonable request. The analysis code, trained model parameters, and documentation are available upon reasonable request.
